# Circulating AIM as an Indicator of Liver Damage and Hepatocellular Carcinoma in Humans

**DOI:** 10.1371/journal.pone.0109123

**Published:** 2014-10-10

**Authors:** Tomoko Yamazaki, Mayumi Mori, Satoko Arai, Ryosuke Tateishi, Masanori Abe, Mihoko Ban, Akemi Nishijima, Maki Maeda, Takeharu Asano, Toshihiro Kai, Kiyohiro Izumino, Jun Takahashi, Kayo Aoyama, Sei Harada, Toru Takebayashi, Toshiaki Gunji, Shin Ohnishi, Shinji Seto, Yukio Yoshida, Yoichi Hiasa, Kazuhiko Koike, Ken-ichi Yamamura, Ken-ichiro Inoue, Toru Miyazaki

**Affiliations:** 1 Laboratory of Molecular Biomedicine for Pathogenesis, Center for Disease Biology and Integrative Medicine, Faculty of Medicine, The University of Tokyo, Tokyo, Japan; 2 Department of Gastroenterology, Graduate School of Medicine, The University of Tokyo, Tokyo, Japan; 3 Department of Gastroenterology and Metabology, Ehime University Graduate School of Medicine, Ehime, Japan; 4 Shunkaikai, Inoue Hospital, Nagasaki, Japan; 5 Department of Gastroenterology, Jichi Medical University, Saitama Medical Center, Omiya, Japan; 6 Department of Preventive Medicine and Public Health, School of Medicine, Keio University, Tokyo, Japan; 7 Center for Preventive Medicine, NTT Medical Center Tokyo, Tokyo, Japan; 8 National Center for Global Health and Medicine, Tokyo, Japan; 9 Center for Animal Resources and Development, Kumamoto University, Kumamoto, Japan; 10 Max Planck-The University of Tokyo Center for Integrative Inflammology, Tokyo, Japan; 11 CREST, Japan Science and Technology Agency, Tokyo, Japan; The Ohio State University, United States of America

## Abstract

**Background:**

Hepatocellular carcinoma (HCC), the fifth most common cancer type and the third highest cause of cancer death worldwide, develops in different types of liver injuries, and is mostly associated with cirrhosis. However, non-alcoholic fatty liver disease often causes HCC with less fibrosis, and the number of patients with this disease is rapidly increasing. The high mortality rate and the pathological complexity of liver diseases and HCC require blood biomarkers that accurately reflect the state of liver damage and presence of HCC.

**Methods and Findings:**

Here we demonstrate that a circulating protein, apoptosis inhibitor of macrophage (AIM) may meet this requirement. A large-scale analysis of healthy individuals across a wide age range revealed a mean blood AIM of 4.99±1.8 µg/ml in men and 6.06±2.1 µg/ml in women. AIM levels were significantly augmented in the younger generation (20s–40s), particularly in women. Interestingly, AIM levels were markedly higher in patients with advanced liver damage, regardless of disease type, and correlated significantly with multiple parameters representing liver function. In mice, AIM levels increased in response to carbon tetrachloride, confirming that the high AIM observed in humans is the result of liver damage. In addition, carbon tetrachloride caused comparable states of liver damage in AIM-deficient and wild-type mice, indicating no influence of AIM levels on liver injury progression. Intriguingly, certain combinations of AIM indexes normalized to liver marker score significantly distinguished HCC patients from non-HCC patients and thus could be applicable for HCC diagnosis.

**Conclusion:**

AIM potently reveals both liver damage and HCC. Thus, our results may provide the basis for novel diagnostic strategies for this widespread and fatal disease.

## Introduction

Chronic liver injury is one of the most common and fatal diseases in modern society. It has multiple causes including hepatitis virus infection mostly due to hepatitis C virus (HCV) and to a lesser extent hepatitis B virus (HBV), alcohol injury, autoimmunity, and genetic disorders such as hemochromatosis [1]–[3]. In addition, the non-alcoholic fatty liver disease (NAFLD), which is associated with obesity, has been observed in a rapidly growing number of patients due to recent and drastic changes in lifestyle. NAFLD comprises a wide variety of disease criteria ranging from benign simple steatosis to progressive inflammation and fibrosis, called non-alcoholic steatohepatitis (NASH) [4], [5]. Such chronic liver diseases exhibit continuous inflammation and fibrosis and are a prominent risk for the development of hepatocellular carcinoma (HCC) [6]–[8]. In contrast to patients with HCV infection, who display a high susceptibility to HCC, only a limited proportion of NAFLD patients progress to carcinoma [9]–[11]. Intriguingly, recent evidence has revealed that although HCC develops largely on the basis of severe liver fibrosis/cirrhosis, it often occurs without cirrhosis in NAFLD/NASH patients exhibiting mild inflammation and fibrosis [12]–[18]. However, the mechanism of how each pathological background induces HCC remains to be elucidated. With such increasing risks and complicated pathogenesis, biomarkers that reflect the state of liver damage and the presence of HCC are important, particularly for the early diagnosis of HCC development. Ideally, markers that indicate an individual's susceptibility to HCC may be desirable from the prognostic and preventive views of HCC.

The circulating protein, apoptosis inhibitor of macrophage (AIM), also called CD5L, was initially identified as an apoptosis inhibitor that supports macrophage survival [19]. AIM is produced solely by tissue macrophages under transcriptional regulation by nuclear receptor liver X receptor alpha (LXRα) [20]–[22], and as a secreted molecule, AIM is detected in both human and mouse blood [19], [23]. Interestingly, AIM associates with the immunoglobulin (Ig)M pentamer in the blood, and this association protects AIM from renal excretion, thereby maintaining circulating AIM at a relatively high concentration (approximately 2–5 µg/ml) in mice [23], [24]. However, AIM's precise levels in healthy individuals and patients with various diseases remain controversial [25]–[28].

We recently identified that AIM is incorporated into adipocytes via CD36-mediated endocytosis where it inactivates cytoplasmic fatty acid synthase (FASN) through direct binding. This response reduces the production of lipid droplet-coating proteins such as fat-specific protein 27 (FSP27) and perilipin, thereby decreasing triacylglycerol deposition within adipocytes [29], [30]. Consistent with this effect, adipocyte hypertrophy was found to be more advanced with a greater mass of visceral adipose tissue in AIM-deficient (*AIM^-/-^*) mice than in wild-type (*AIM^+/+^*) mice fed a high-fat diet (HFD) [29]. We also found that AIM prevents lipid storage in the liver, as in adipocytes [31]. Because a consensus has rapidly emerged that hepatocytic lipid metabolism impacts the pathogenesis of not only NAFLD but also other liver injuries, as well as HCC development, we decided to address the possible relationship in circulating AIM levels, the state of liver damage, and the presence of HCC in humans.

In this study, we first analyzed a large number of healthy individuals to determine the “normal level” of circulating AIM. We then assessed the correlation between circulating AIM levels and the state of liver damage using sera from patients with liver diseases. We also tested whether the difference in AIM levels correlated with the progression of liver damage using a mouse model. Furthermore, we investigated whether the AIM level can be applied for the diagnosis of HCC in humans.

## Methods

### Human subjects

Serum samples of healthy individuals were collected from volunteers who had annual medical examinations at Inoue Hospitals (Nagasaki, Japan). Serum samples of patients with liver diseases were obtained from Tokyo University Hospital, Ehime University Hospital and Jichi Medical University Hospital.

### Ethic

For analysis of human subjects, informed consent in writing was obtained from each healthy volunteer and patient, and the study protocol conformed to the ethical guidelines of the 1975 Declaration of Helsinki as reflected in a priori approval by the Ethics Committee of the University of Tokyo for Medical Experiments (Permission Numbers: #3358 & #2817). In addition, all animal experiments were carried out in strict accordance with the recommendations in the Guide for the Care and Use of Laboratory Animals of the National Institutes of Health. The protocol was approved by the Committee on the Ethics of Animal Experiments of the University of Tokyo (Permit Number: P10-143). All surgery was performed under sodium pentobarbital anesthesia, and all efforts were made to minimize suffering.

### Carbon tetrachloride (CCl4) administration

*AIM^-/-^* mice [19] had been backcrossed to C57BL/6 (B6) for 15 generations before used for experiments. Mice were intraperitoneally injected with CCl4 (Wako, Osaka, Japan) (1.6 g/kg body weight; dissolved in corn oil) twice a week for 3 or 12 wk. Mice were sacrificed 3 days after the last injection of CCl_4_. All mice were maintained under a specific pathogen-free (SPF) condition.

### Statistical analysis

Student's *t*-test was performed to compare values from two groups. Correlation coefficients and *p* values were calculated by Excel. Multiple linear regression analysis was performed by backward stepwise approach, with t>1.5 for entry and t<1.5 or inter-variables correlation coefficient>0.5 or probability F>0.1 for removal from the model. Multiple pairwise comparison among groups were performed by ANCOVA using JMP software (version 11).

### ELISA assay

Human AIM was measured by an ELISA system using mouse anti-human AIM monoclonal antibodies (clones #6 and #7; established in our laboratory), which is now available from the Trans Genic Inc., Kumamoto, Japan. For ELISA of mouse AIM, we used two different rat anti-mouse AIM monoclonal antibodies (clones #36 and #35; established in our laboratory). Human IgM was measured by Human IgM ELISA Quantification Set (Bethyl Laboratories, Inc. Montgomery, USA).

### Histology

Liver specimens were fixed overnight in 4% paraformaldehyde in phosphate buffered saline (PBS) and replaced into 30% sucrose/PBS liquid. Samples were embedded in Tissue-Tek O.C.T. compound (Sakura Finetek Co.,Ltd., Tokyo), cut by 10 µm. For Sirius red staining, sections were washed in PBS for 5 min, counter stained with Mayer's Hematoxylin for 10 min, washed with running water for 2 min and subsequently soaked in hydrochloric acid alcohol (0.5% HCl in 70% EtOH) for 1 min. Sections were then stained with 0.03% Sirius red (Direct red 80, SIGMA-ALDRICH) in saturated picric acid solution for 15 min. HE staining was performed using Mayer's Hematoxylin (MUTO PURE CHEMICALS CO.,LTD., Tokyo) and Eosin (SIGMA-ALDRICH, St. Louis, USA).

### Fibrosis analysis

Fibrosis area determined by Sirius red staining was quantified using NIH Image J software. Five areas for each sample were assessed under a microscope (FSX 100, OLYMPUS, Tokyo).

### Quantitative PCR assay

The quantitative evaluation of mRNA was performed by the ΔΔC_T_ method using a 7500Fast Real-Time PCR system (Life Technologies Japan, Tokyo) and Power SYBR Green PCR Master Mix (Life Technologies). Sequences of the oligonucleotides used are below:

f-GAPDH 5′-AACTTTGGCATTGTGGAAGG-3′

r-GAPDH 5′-GGATGCAGGGATGATGTTCT-3′

f-TNFα  5′-ACGGCATGGATCTCAAAGAC-3′

r-TNFα  5′-AGATAGCAAATCGGCTGACG-3′

f-IL1β   5′-CTGGTGTGTGACGTTCCCATTA-3′

r-IL1β   5′-CCGACAGCACGAGGCTTT-3′

f-IL 6   5′-CCAGTTGCCTTCTTGGGACT-3′

r-IL 6   5′-GGTCTGTTGGGAGTGGTATCC-3′

f-MCP1  5′-ACTGAAGCCAGCTCTCTCTTCCTC-3′

r-MCP1  5′-TTCCTTCTTGGGGTCAGCACAGAC-3′

f-CD11c 5′-GAGCCAGAACTTCCCAACTG-3′

r-CD11c 5′-TCAGGAACACGATGTCTTGG-3′

f-CD163 5′-CCTGGATCATCTGTGACAACA-3′

r-CD163 5′-TCCACACGTCCAGAACAGTC-3′

f-Arg-1  5′-CTCCAAGCCAAAGTCCTTAGAG-3′

r-Arg-1  5′-AGGAGCTGTCATTAGGGACATC-3′

f-MR   5′-CCACAGCATTGAGGAGTTTG-3′

r-MR   5′-ACAGCTCATCATTTGGCTCA-3′

f-TGF β 5′-TGGAGCAACATGTGGAACTC-3′

r-TGF β 5′-CAGCAGCCGGTTACCAAG-3′

f-α SMA 5′-ACTCTCTTCCAGCCATCTTCA-3′

r-α SMA 5′-ATAGGTGGTTTCGTGGATGC-3′

f-Col4a1 5′-TTAAAGGACTCCAGGGACCAC-3′

r-Col4a1 5′-CCCACTGAGCCTGTCACAC-3′

f-CTGF 5′-TGACCTGGAGGAAAACATTAAGA-3′

r-CTGF 5′-AGCCCTGTATGTCTTCACACTG-3′

f-TIMP1 5′-GCAAAGAGCTTTCTCAAAGACC-3′

r-TIMP1 5′-AGGGATAGATAAACAGGGAAACACT-3′

f-mAIM 5′-GAGGACACATGGATGGAATGT-3′

r-mAIM 5′-ACCCTTGTGTAGCACCTCCA-3′

## Results

### Circulating AIM levels in healthy individuals

**Figure 1:**
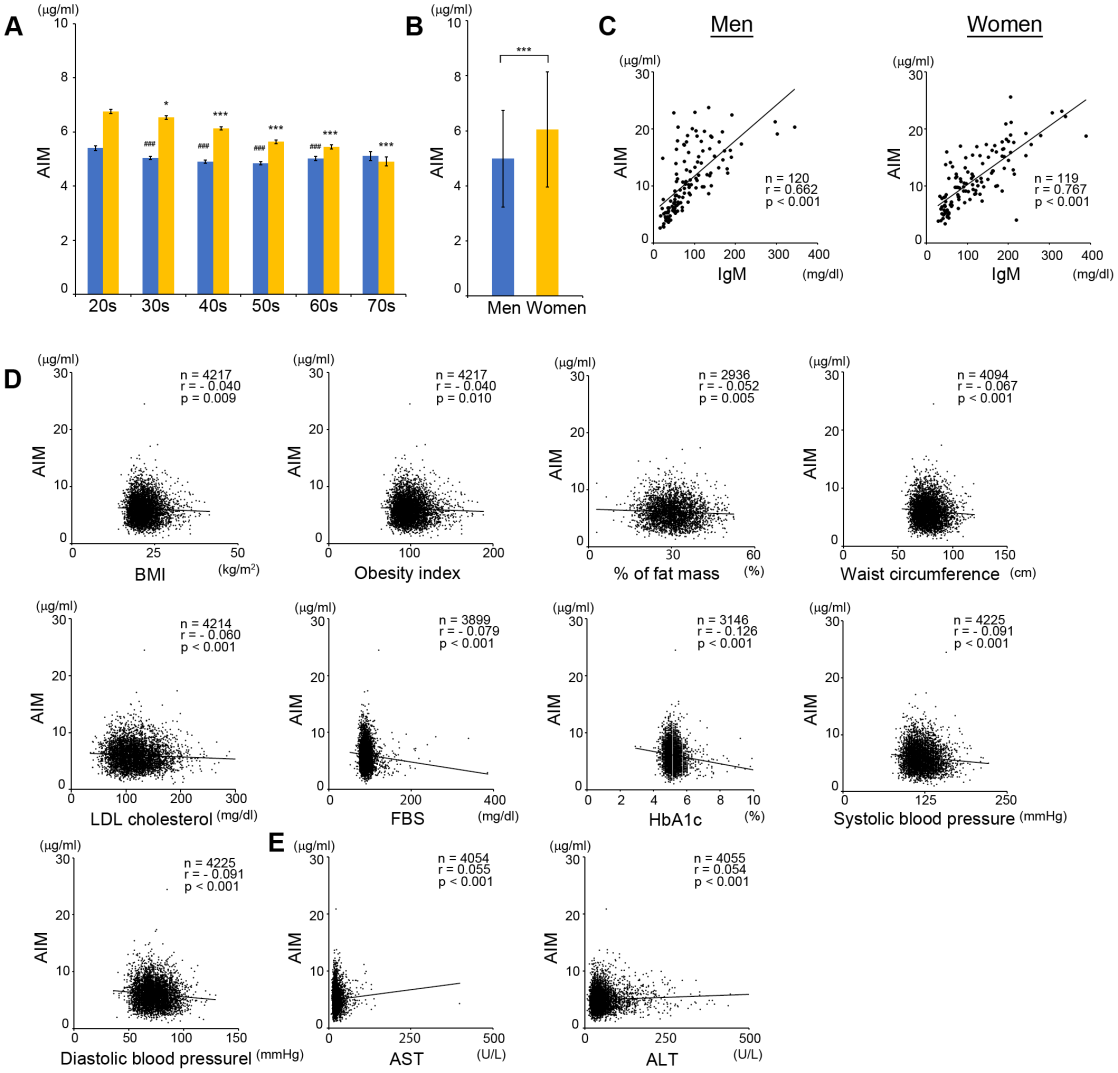
Circulating AIM levels in healthy indivisuals. (A) AIM levels in different generations. Error bar: SEM. ***: *p*<0.001 vs. the value of women in 20s. ###: *p*<0.001 vs. the value of men in 20s. (B) Means ± SD (µg/ml) of AIM levels in whole men and women. AIM levels were significantly higher in women than in men. (C) Correlation of AIM and IgM levels in men and women. IgM levels were analyzed by ELISA in 20 individuals exhibiting a variety of AIM levels in each generation in men and women. (D) Correlation in AIM levels and BMI, obesity index, % of fat mass or waist circumference, LDL cholesterol levels, HbA1C, FBS, systolic or diastolic blood pressure in women. (E) Correlation in AIM and AST or ALT levels in males and females. In C-E, r: correlation coefficients in single linear regression analysis, p: p value, n: number of samples. Blue dots: men, yellow dots: women.

To investigate circulating AIM levels in healthy individuals, we performed a large-scale analysis of AIM using more than 8,000 blood samples of volunteers attending annual medical examinations in 2012 and 2013. For this study, we established an ELISA system by generating monoclonal antibodies that accurately estimated human AIM levels in blood. The composition of volunteers and the mean±SD AIM level (µg/ml) are shown in Table 1. AIM levels were highest in both men and women in their 20s and decreased with age (Fig. 1A). In individuals <50 years old, AIM levels were significantly higher in women (Fig. 1A), resulting in an overall higher mean AIM level in women (Fig. 1B). Consistent with our previous report [23], a strong correlation was observed between IgM and AIM levels (Fig. 1C).

**Table 1:** The composition of examinees and the AIM level.

	Male		Female	
age	AIM (µg/ml)	n	AIM (µg/ml)	n
10s	5.62±1.66	28	6.39±1.65	15
20s	5.41±1.67	368	6.75±2.05	592
30s	5.04±1.69	806	6.53±2.03	791
40s	4.90±1.76	1153	6.14±2.09	1163
50s	4.84±1.78	988	5.64±1.95	977
60s	5.02±1.80	580	5.45±2.1	579
70s	5.11±1.79	120	4.91±1.57	87
80s	4.69±1.57	9	5.57±2.84	16
90s	7.55±3.60	3	4.22±2.03	5
whole	4.99±1.76	4055	6.06±2.09	4225

The AIM level is presented as mean±SD (µg/ml). n: sample number.

The significance of the relationships between AIM levels and various clinical parameters is presented in Table S1. In particular, we focused on obesity-related parameters because AIM has lipolytic function and thus acts as an anti-obese factor in mice [29], [30]. The relationship between AIM and various parameters was more significant in women, and AIM levels correlated negatively with body mass index, obesity index, % fat mass, and waist circumference (Fig. 1D). In line with these results, AIM levels also correlated negatively with low-density lipoprotein (LDL) cholesterol levels (Fig. 1D) and several diabetic markers including fasting blood sugar (FBS) and glycated hemoglobin (HbA1C) (Fig. 1D), as well as with blood pressure, in women (Fig. 1D). Thus, consistent with the findings of our previous study in mice [29], [30], AIM levels correlated negatively with multiple parameters related to obesity, and these correlations were more prominent in women. Intriguingly however, a weak but significant positive correlation was found between AIM levels and biomarkers of hepatocyte injury, including aspartate aminotransferase (AST) and alanine aminotransferase (ALT), particularly in men (Fig. 1E). Taken together, it is likely that AIM levels increase along with the progression of liver injury. In both men and women, a significant negative correlation was unexpectedly found between AIM levels and red blood cell numbers, but the reason underlying this correlation is unclear (Table S1).

### AIM levels correlated strongly with liver function in liver injury

We next analyzed blood samples from patients with chronic hepatitis and liver cirrhosis. As depicted in Table 2, the cause of liver injury in the majority of the patients was hepatitis virus infection, whereas non-infected cases constituted a lower proportion of the patients with alcoholic liver failure and NAFLD/NASH. Patients with or without HCC were investigated. The clinical features of the patients tested are presented in Table 3 focusing on the liver function.

**Table 2:** Number of patients in each type of liver injury.

	HCC		Non HCC	
	Men	Women	Men	Women
**Whole**	189 (100%)	86 (100%)	90 (100%)	56 (100%)
**HBV**	36 (19%)	6 (7%)	28 (31%)	14 (25%)
**HCV**	116 (61%)	61 (71%)	30 (33%)	26 (47%)
**HBV and HCV**	1 (1%)	1 (1%)	0 (0%)	0 (0%)
**Alcoholic hepatitis**	21 (11%)	2 (2%)	4 (5%)	3 (5%)
**NAFLD**	0 (0%)	0 (0%)	21 (23%)	8 (14%)
**NASH**	3 (2%)	5 (6%)	6 (7%)	4 (7%)
**Cryptogenic or other**	12 (6%)	11 (13%)	1 (1%)	1 (2%)

The percentage shows the proportion in total number of each gender with or without HCC.

**Table 3:** Clinical features of patients analyzed for AIM.

	HCC		Non HCC	
	Men	Women	Men	Women
**Age (year)**	66±10 [32–87]	70±9 [33–87]	55±15 [23–84]	62±13 [31–80]
**AIM (µg/ml)**	5.7±2.8 [1.7–18.6]	5.8±2.6 [0.8–14.7]	5.1±2.7 [1.5–17.5]	5.8±2.9 [1.8–16.9]
**IgM (mg/dl)**	144.8±101 [24.2–708.0]	143.4±98.0 [1.6–769.2]	103.3±49.4 [18.7–216.4]	129.4±64.1 [42.8–346.0]
**AST (U/L)**	56.3±30.7 [14–170]	59.6±42.9 [17–312]	51.8±43.3 [13–287]	44.7±24.5 [14–139]
**ALT (U/L)**	53.6±37.6 [8–233]	48.0±39.5 [9–315]	60.3±66.8 [6–498]	40.4±26.6 [11–121]
**TB (mg/dl)**	0.98±0.54 [0.3–3.9]	1.02±0.61 [0.4–3.8]	1.13±0.85 [0.3–5.1]	1.07±1.17 [0.4–9.2]
**DB (mg/dl)**	0.35±0.32 [0.1–0.7]	0.38±0.32 [0.1–1.6]	0.29±0.28 [0.1–1.6]	0.25±0.13 [0.1–0.5]
**ALB (g/dl)**	3.69±0.46 [2.3–4.7]	3.64±0.49 [2.2–4.6]	4.06±0.49 [2.5–5.2]	3.89±0.58 [2.2–4.5]
**PLT (x10^4^/mm^3^)**	12.3±5.29 [3.0–28.3]	11.1±5.43 [4.2–31.9]	16.6±7.32 [2.1–35.6]	13.9±6.57 [2.3–26]
**PT (%)**	85.4±14.3 [53–100]	84.3±14.7 [42–100]	87.1±17.2 [28–100]	84.0±19.7 [32–100]
**Cre (mg/dl)**	0.81±0.19 [0.40–1.56]	0.61±0.14 [0.4–1.04]	0.83±0.20 [0.54–1.95]	0.66±0.26 [0.49–2.21]
**ICG (%)**	23.8±14.4 [2.7–72.4]	24.7±15.6 [0.9–71.2]	-	-

The mean ± SD as well as the range of diversity are presented for each parametric variable. The ICG score was only available in HCC patients.

**Figure 2:**
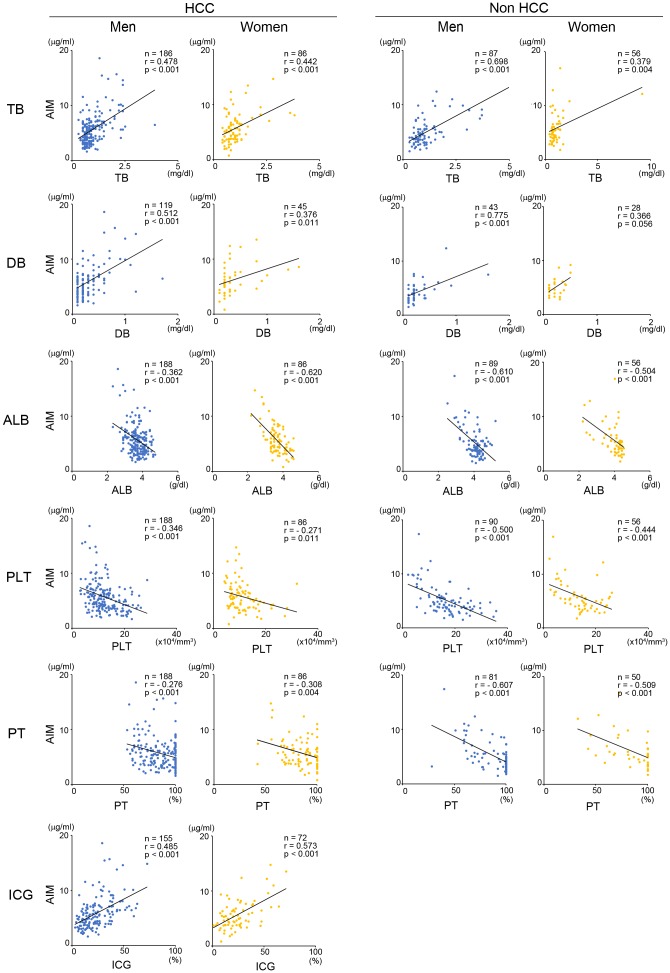
Correlation in the AIM level and the liver function under liver injury. Correlation in AIM levels and various biomarkers representing liver function in men (blue) and women (yellow), with or without HCC. ICG score was only available in HCC patients.

**Figure 3:**
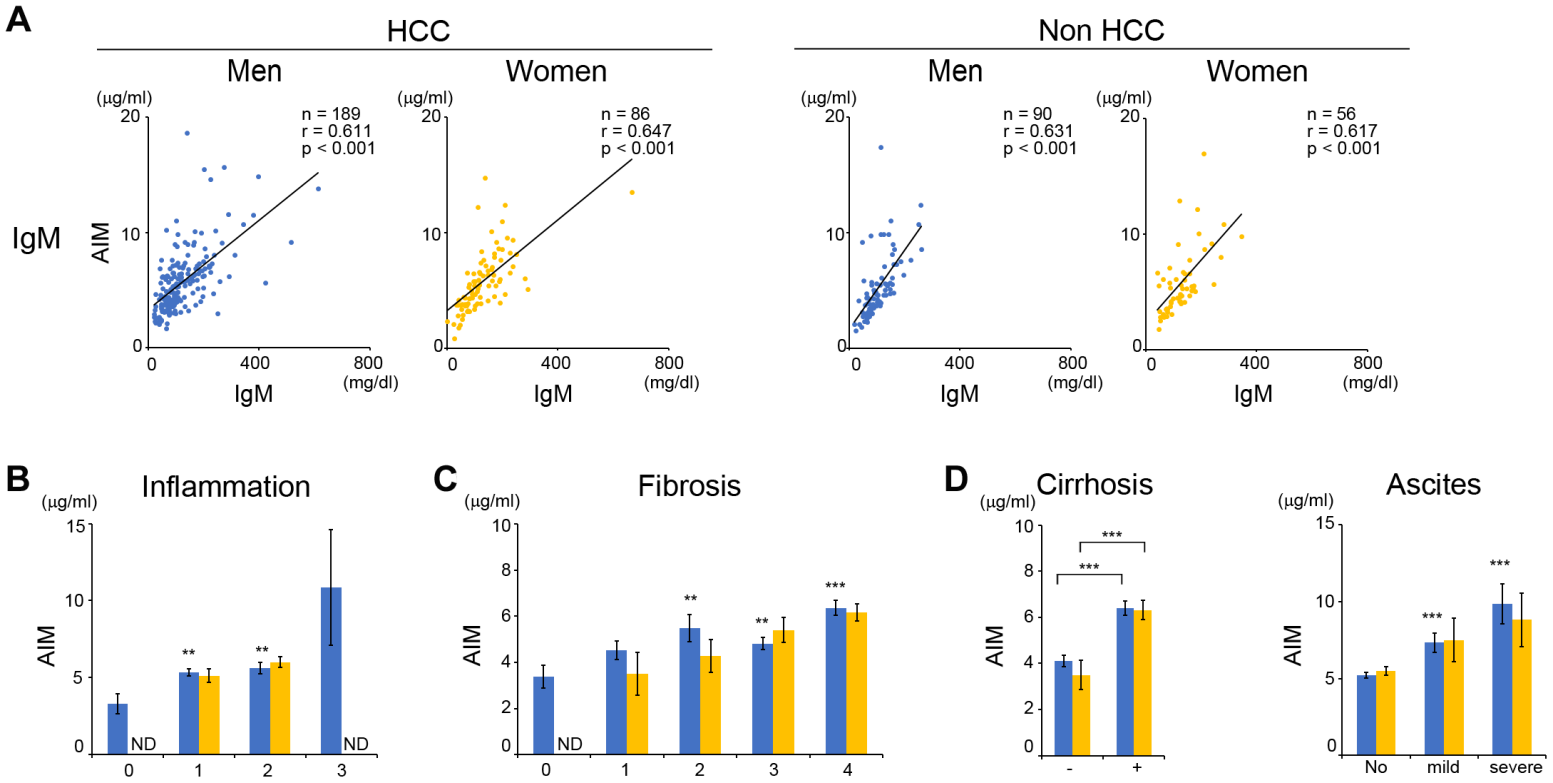
The AIM level increases with progression of liver inflammation and fibrosis. (A) Correlation in AIM and IgM levels in men (blue) and women (yellow), with or without HCC. (B) AIM levels in HCC patients with different inflammatory levels according to the Inuyama classification. (C) AIM levels in HCC patients with different fibrotic scores according to the Inuyama classification. (D) AIM levels in HCC patients with different grades of cirrhosis or ascites. In C-E, AIM levels are presented as means±SEM (µg/ml).

The positive correlation between AIM levels and AST/ALT for liver injury, which was already seen in individuals without severe liver damage (Fig. 1E) was notably more obvious in men and women with advanced liver damage. Highly significant correlations were observed between AIM and multiple biomarkers, thereby reflecting liver function, including total or direct bilirubin (TB or DB), albumin (ALB), platelet count (PLT), % prothrombin time (%PT), and the indocyanine green (ICG) test (Fig. 2). These correlations were obvious in individuals with or without hepatitis virus infection (Table S2) and in the presence or absence of HCC (Fig. 2). In HCC patients, there was no significant correlation in levels of AIM and several HCC markers including alpha fetoprotein (AFP), des-gamma-carboxyprothrombin (DCP, also called prothrombin induced by vitamin K-absence II; PIVKA-II), and AFP fraction L3 (L3) (Fig. S1). As in healthy individuals, a significant correlation was also found between AIM and IgM levels in patients with liver injury (Fig. 3A); namely, IgM levels increased with the progression of liver damage (Fig. S2).

We then addressed which parameters correlated independently with AIM levels. To this end, we performed multiple regression analysis by the backward stepwise method including AIM. Table S3 shows the correlation coefficients between all parameters that reflect liver function and thus were candidates for determinant of AIM level. When confounding factors were eliminated, IgM, TB, ALB, and %PT in men (R^2^ = 54.0%) and IgM, TB, and ALB in women (R^2^ = 57.0%) were independent determinants of AIM in HCC patients (Table 4). In non-HCC patients, IgM, ALB, and PLT in men (R^2^ = 63.4%) and IgM and PLT in women (R^2^ = 53.6%) were the independent determinants of AIM (Table 4). In particular, in men, the t-value of TB in HCC patients was 5.39, but was not significant in non-HCC patients. In women, the t-value of ALB was −4.80 in HCC patients, but again was not significant in non-HCC patients (Table 4).

**Table 4:** Multiple linear regression analysis.

	HCC Men (n = 173) Non-HCC Men (n = 89)					
	C	*t*	*p*	C	*t*	*p*
R^2^	53.98%			63.44%		
Intercept	5.30	2.927	0.004	10.2	6.29	<0.001
IgM	0.0137	9.37	<0.001	0.0266	7.33	<0.001
TB	1.60	5.39	<0.001	―	―	―
ALB	−0.886	−2.53	0.012	−1.41	−3.59	<0.001
PLT	―	―	―	−0.127	−4.88	<0.001
PT	−0.0191	−1.77	0.079	―	―	―

	HCC Women (n = 86)			Non-HCC Women (n = 56)		
	C	*t*	*p*	C	*t*	*p*
R^2^	57.02%			53.62%		
Intercept	9.58	4.82	<0.001	4.88	5.90	<0.001
IgM	0.0104	4.66	<0.001	0.0265	6.48	<0.001
TB	0.862	2.48	0.016	―	―	―
ALB	−2.17	−4.80	<0.001	―	―	―
PLT	―	―	―	−0.181	−4.53	<0.001

Independent determinants for AIM in men or women with or without HCC. R^2^: determination coefficient, C: regression coefficient, *t*: t-value, *p*: p-value, in multiple linear regression models. ―: not independent.

Similar results were obtained for the relationship between AIM and the grade of liver inflammation or fibrosis. Levels in inflammation (0–3) and fibrosis (F0-F4) were evaluated according to the Inuyama classification [32]. The number of patients in each category is presented in Table 5. Clinical information about inflammation and fibrosis were not available in a part of HCC patients and all of non-HCC patients. AIM levels were higher in those with liver inflammation (Fig. 3B). AIM also increased in line with the progression of liver fibrosis (Fig. 3C). Similarly, AIM levels were higher in patients with cirrhosis or ascites (Fig. 3D). Note that all cirrhotic patients tested in this study were under a compensated stage. Since some patients exhibited prominently high or low levels of AIM (>10 µg/ml or <3.0 µg/ml), we assessed relationship in levels of AIM and various parameters in these populations. However, there was no remarkable correlation in AIM and any parameter (Table S4). No significant correlation was observed between AIM levels and alcohol intake (data not shown).

**Table 5:** Number of patients showing different levels of inflammation, fibrosis, cirrhosis, or ascites.

		Men	Women
**Whole**		189	86
**Inflammation (in %)**	0	6 (3%)	0 (0%)
	1	88 (47%)	36 (42%)
	2	50 (26%)	28 (32%)
	3	2 (1%)	0 (0%)
	unknown	43 (23%)	22 (26%)
**Fibrosis (in %)**	0	7 (4%)	0 (0%)
	1	14 (7%)	3 (3%)
	2	19 (10%)	13 (15%)
	3	42 (22%)	11(13%)
	4	81 (43%)	42 (49%)
	unknown	26 (14%)	17 (20%)
**Cirrhosis (in %)**	-	36 (19%)	6 (7%)
	+	82 (43%)	45 (52%)
	unknown	71 (38%)	35 (41%)
**Ascites (in %)**	No	159 (84%)	77 (90%)
	Mild (1∼2L)	18 (10%)	5 (6%)
	Severe (3∼5L)	10 (5%)	4 (4%)
	unknown	2 (1%)	0 (0%)

All patients here possessed HCC. The percentage shows the proportion in each level of identical phenotype. Unknown: Information was not available.

### AIM*^−/−^* mice had comparable liver damage to wild-type mice in response to CCl4

**Figure 4:**
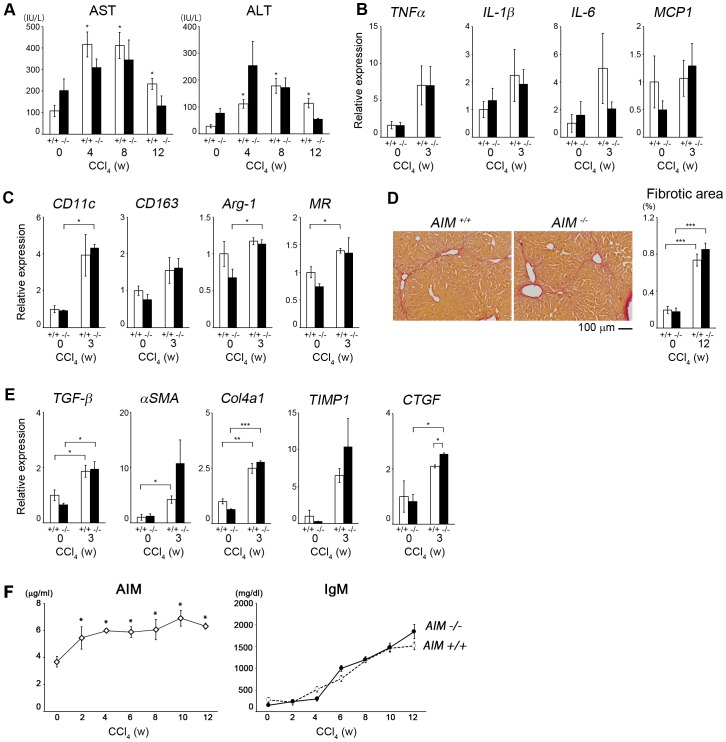
*AIM^+/+^* and *AIM^-/-^* mice exhibit comparable liver damage in response to CCl_4_. (A) AST and ALT at 0, 4, 8, 12 wk after administration of CCl_4_ (1.6 g/kg body weight, twice injection per week for 12 weeks) in *AIM^+/+^* mice (+/+) and *AIM^-/-^* mice (−/−). n = 3 for each. Error bar: SEM, *: p<0.05 vs. before CCl4 administration (0 w). (B, C) mRNA levels of *TNFα*, *IL-1β*, *IL-6* and *MCP-1* (B); or *CD11c*, *CD163*, *Arg-1* and *MR* (C) were assessed by QPCR using RNA isolated from liver after administration of CCl_4_ for 3 weeks. n = 3 for each. Error bar: SEM, *: *p*<0.05. (D) Sirius-red staining of the liver specimens after administration of CCl_4_ for 12 weeks to *AIM^+/+^* mice (+/+) and *AIM^-/-^* mice (−/−). Bar: 100 µm. Right graph shows the quantification of fibrotic area. (E) mRNA levels of *TGFβ*, *αSMA*, *Col4a1* and *CTGF* were assessed by QPCR using RNA isolated from liver from mice after administration of CCl_4_ for 3 weeks. n = 3 for each. Error bar: SEM; *: *p*<0.05, ***: *p*<0.001. (F) *Left*: Serum AIM levels were measured by ELISA from wild-type mice after administration of CCl_4_. n = 6 for each. Error bar: SEM, *: *p*<0.05 vs. before CCl_4_ administration (0 w). *Right*: Serum IgM levels were measured by semi-quantitative immunoblotting using sera from *AIM^+/+^* (+/+) mice and *AIM^-/-^* (−/−) after administration of CCl_4_. Purified mouse IgM clone (3F3) was used as standard. Quantification of signals from immunoblotting was performed by using ImageQuant TL software (GE Healthcare, Little Chalfont, UK). n = 6 for each. Error bar: SEM; *: *p*<0.05, ***: *p*<0.01, vs. before CCl_4_ administration (0 w) in *AIM^+/+^* (+/+) mice; ###: *p*<0.001 vs. before CCl_4_ administration in *AIM^-/-^* mice.

Next, we assessed whether high AIM levels directly promote liver injury or whether AIM levels increase as a result of liver damage progression. To this end, we employed animal models of progressive liver injury induced by carbon tetrachloride. *AIM^-/-^* and *AIM^+/+^* mice were challenged with CCl4 (1.6 g/kg body weight) injected twice a week for 12 weeks, and the state of liver injury was assessed. As demonstrated in Fig. 4A, AST and ALT levels were similar at multiple time points in *AIM^+/+^* and *AIM^-/-^* mice, indicating that the liver was comparably damaged in the presence or absence of AIM. Note that the average of circulating AIM levels without CCl4 administration was 3.3 µg/ml in both males and females. Inflammatory states were investigated during the early phase (after 3 weeks of CCl4 challenge) by measuring mRNA levels for various pro-inflammatory cytokines using quantitative RT-PCR (QPCR). No significant differences were observed in the increased expression levels of *TNFα*, *IL-1β*, *IL-6*, and *MCP-1* in both types of mice (Fig. 4B). In line with this result, similar expression profiles of the M1 and M2 macrophage marker genes (*CD11c* for M1; *CD163*, *Arg-1*, and *mannose receptor* (*MR*) for M2) were observed in *AIM^+/+^* and *AIM^-/-^* mice (Fig. 4C), suggesting that the absence of AIM did not influence the activation state or the M1/M2 polarity of liver macrophages in response to carbon tetrachloride, resulting in comparable liver inflammation progression in both types of mice. Consistent with this finding, liver fibrosis progressed comparably in *AIM^-/-^* and *AIM^+/+^* mice, and Sirius-red staining of liver specimens showed a similar increase in fibrotic areas in both types of mice (Fig. 4D). Accordingly, mRNA levels of various markers of fibrosis progression such as *TGFβ*, *αSMA*, *Col4a1*, and *connective tissue growth factor* (*CTGF*) were also comparable in *AIM^-/-^* and *AIM^+/+^* mice (Fig. 4E). Taken together, these results clearly indicate that the presence or absence of AIM did not influence the state of liver injury in response to carbon tetrachloride. Thus, it is likely that the augmented AIM levels observed in humans (Fig. 2) were the result of liver damage. Further supporting this notion is the finding that AIM levels increased markedly with progression of liver damage in *AIM^+/+^* mice (Fig. 4F, left). However, AIM mRNA levels in the *AIM^+/+^* liver did not increase in response to CCl4 (Fig. S3), suggesting that the increase in blood AIM was not brought about by enhancement of AIM production in liver Kupffer macrophages, one of the highest AIM-producing cell types [19].

Interestingly, no significant difference in increase in IgM levels was observed in both *AIM^-/-^* and *AIM^+/+^* mice, suggesting that the increase in IgM was independent of AIM (Fig. 4F, right). This result is reminiscent of our previous finding of a similar increase in IgM levels in *AIM^-/-^* and *AIM^+/+^* mice in response to a HFD [23].

### Diagnostic application of AIM for HCC

**Figure 5:**
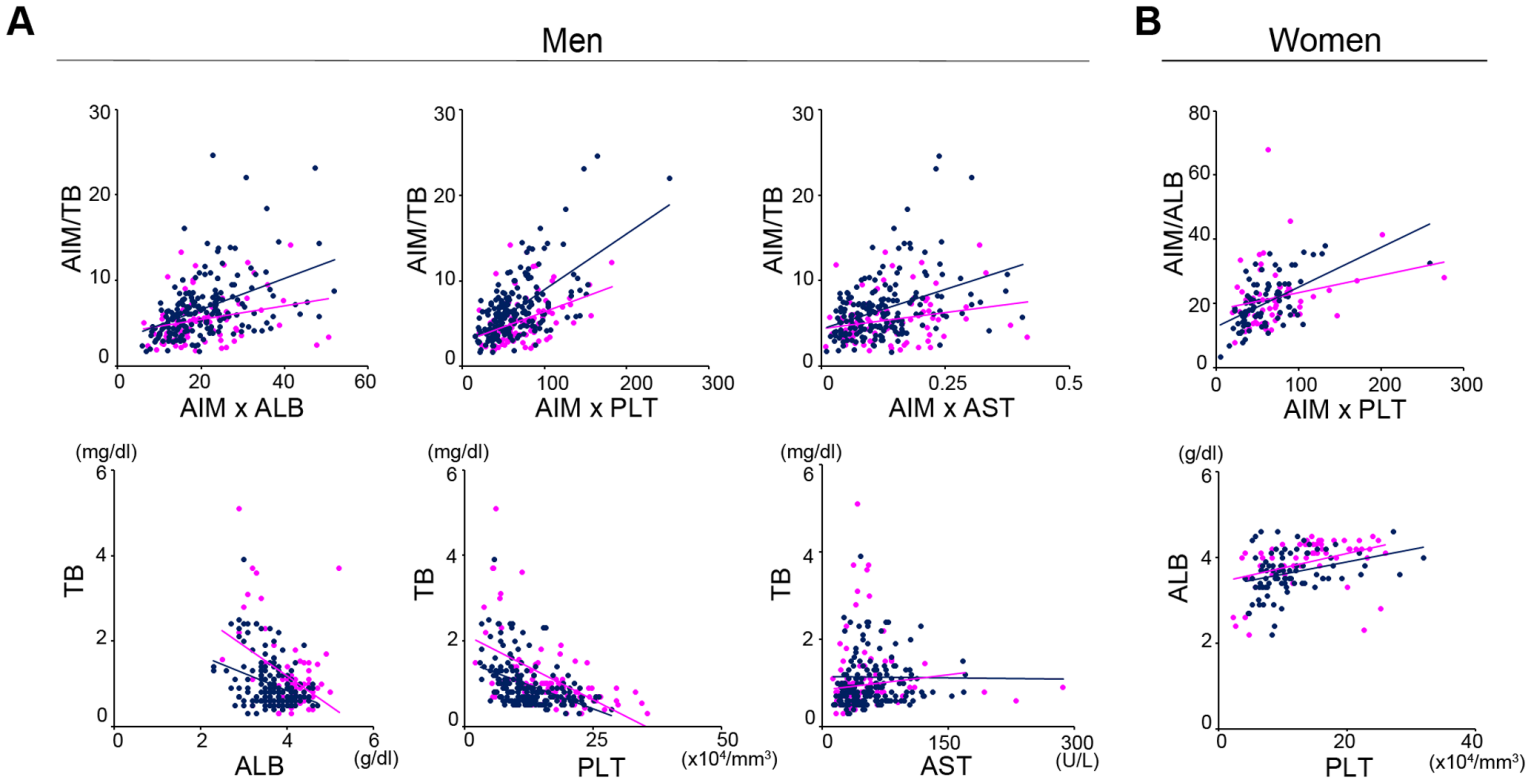
AIM-index distinguishes HCC and non-HCC patients. (A) AIM-TB index vs. AIM-ALB index, AIM-PLT index or AIM-AST index in men with or without HCC. TB vs. ALB, PLT or AST are also presented. (B) AIM-ALB index vs. AIM-%PT index in women with or without HCC. ALB vs. %PT is also presented. r: correlation coefficients; p: p value determined by ANCOVA. Blue dots and bars: HCC patients, red dots and bars: non-HCC patients.

As demonstrated in Fig. 2, AIM levels increased in line with the progression of liver injury in patients with or without HCC. We then wondered whether HCC and non-HCC patients who show an equivalent score of certain liver biomarkers exhibited different AIM levels. Therefore, we normalized the level of AIM (AIM index) to that of each biomarker. The AIM level was divided by a biomarker score when both correlated positively (*e.g.* AST, ALT, TB, and DB), whereas the scores were multiplied when AIM and the biomarker correlated negatively (*e.g.* ALB, platelets, and %PT). As depicted in Fig. 5A, the ratio of the AIM-TB index to the AIM-ALB index in men was significantly higher in HCC patients than in non-HCC patients by analysis of covariance (ANCOVA), but no significant differences were observed for the ratio of the TB score to the ALB score between HCC and non-HCC patients. Similar results were obtained for the ratio of the AIM-TB index to the AIM-PLT or AIM-AST index, although TB correlated with neither PLT nor AST (Fig. 5A). In women, a comparison of the AIM-ALB and AIM-%PT indexes revealed similar differences between the HCC and non-HCC patients (Fig. 5B). Note that TB in male HCC patients and ALB in female HCC patients produced high t-values in multiple regression analysis when assessing the determinants of AIM (Table 3).

## Discussion

This study provides the first large-scale description of the circulating AIM levels in the general population and in the context of liver function parameters in humans. A variety of new findings were obtained as follows. Firstly, relatively higher AIM levels were observed in the younger generation, especially in women, suggesting potential involvement of estrogen in the increase in circulating AIM levels. Accumulating evidence of estrogen-associated physiology including suppression of triacylglycerol (TG) storage in fat and liver tissues [33]–[36], reduction of expression and enzymatic activity of FASN [37], and preventive effect for foam cell formation and the development of atherosclerosis [38]–[41], which are all reminiscent of AIM function [20], [29], [30], [42], [43]. Although *AIM* mRNA is expressed under transcriptional regulation by LXR [20]–[22], the impact of estrogen on LXR activation is controversial. For instance, Wang et al. recently reported that E2 activates LXRα [41], whereas suppression of LXRα by E2 was also reported in hepatocytes [34], adipocyte [36], and pancreatic β cells [37]. Alternatively, evidence has shown that estrogen stimulates natural IgM production through B lymphocyte activation [44], [45]. This effect certainly increases AIM levels based on the strong correlation between AIM and IgM. However, further studies are required to clarify the precise involvement of estrogen in the regulation of age-dependent AIM levels in humans. It is noteworthy that in individuals without advanced liver damage, AST and ALT showed a weak positive correlation with AIM levels only in men (Fig. 1E and Table S1). It might be possible that the sex-dependent difference of AIM levels obscured the correlation in women.

Secondly, patients with advanced liver damage exhibited high AIM levels. We postulate that the mean±SD AIM level (µg/ml) in each generation in men and women presented in Table 1 can be defined as the “normal range” of AIM levels. Certainly, however, whether AIM levels that are higher or lower than this range mean pathological may depend on the type of disease. At least, patients with progressive liver damage exhibited significantly higher levels than the normal range. Based on the results of mouse experiments demonstrating that the presence or absence of AIM does not influence the state of liver injury in response to CCl4, and that AIM levels increase in response to CCl4 in wild-type mice, it is likely that AIM levels increase as a result of liver damage. Thus, AIM can be used as a novel biomarker for liver injury. The mRNA level of liver *AIM* did not increase in response to CCl4, indicating no enhanced AIM production by liver Kupffer macrophages. It remains possible, however, that inflammatory stimuli caused by carbon tetrachloride will increase *AIM* expression in macrophages in other tissues such as in the peritoneal cavity and splenic marginal zone, but additional experiments are required to assess this possibility. Alternatively, liver damage might increase AIM stability in the blood. Natural IgM is catabolized mainly in the liver [46]–[48]. Therefore, it is possible that the progression of liver damage prolongs the half-life of IgM, resulting in advanced accumulation of circulating AIM. This scenario can also be applied in humans, and may explain the more profound increase in AIM levels in cirrhotic patients compared with non-cirrhotic patients. A precise assessment of the half-life of IgM and AIM in the presence or absence of liver injury can evaluate this possibility.

More notably, we found that use of the AIM index, which is the blood AIM level normalized to the liver biomarker score, appeared to be useful for distinguishing HCC and non-HCC patients. The AIM-TB index of men with an equivalent AIM-ALB or AIM-PLT index was significantly higher in HCC patients than in non-HCC patients. This is not secondary effect of the correlation in TB and ALB or PLT, as there was no significance in the difference in these correlations in HCC and non-HCC groups. The same conclusion was obtained for the AIM-ALB and AIM-platelets indexes in women. Thus, the presence of HCC may increase the AIM-TB index in men and the AIM-ALB index in women. If this is the case, the AIM index can serve as a novel tumor marker and will be useful for the diagnosis of HCC. Further analysis using HCC-bearing mouse models in the presence or absence of AIM may be appropriate for evaluating this possibility. Alternatively, one could also speculate that individuals who show an enhanced increase in certain AIM indexes (*i.e.* AIM-TB index in men and AIM-ALB index in women) in response to liver injury might be more susceptible to HCC. However, further study such as prospective cohort study of HCC development in patients with similar levels of liver damage and different levels of circulating AIM is certainly needed to assess this possibility. In either case, further investigation will corroborate the applicability of the AIM index for the early detection of HCC.

Since HCC is one of the most common malignant tumors with an increasing incidence, identifying serological biomarkers are extremely needed, especially because most of HCC cases are diagnosed at a late stage. Thus, our study could be the bases of application of circulating AIM level as a diagnostic and/or prognostic marker of HCC, either solo or in combination with other biomarkers.

## Supporting Information

Figure S1 **Correlation between AIM levels and various HCC markers.** Men: blue dots, women: yellow dots. In HCC patients, no significant correlation was observed in levels of AIM and either HCC marker.
(TIF)

Figure S2 **Correlation between IgM levels and various biomarkers representing liver function.** Men: blue dots, women: yellow dots. ICG score was only available in HCC patients.
(TIF)

Figure S3 ***AIM*** **expression did not increase in the liver in response to CCl_4_.** mRNA levels of *AIM* in the liver from wild-type mice after administration of CCl_4_ for 3 wk. n = 3 for each. Error bar: SEM.
(TIF)

Table S1 **Correlation in AIM level and different clinical parameters.** Number of samples, and the correlation coefficients and p values in the correlation with AIM levels in separate tested item. n: sample numbers.
(DOCX)

Table S2 **Correlation between AIM and liver function in patients with or without hepatitis viral infection.** Number of samples, and the correlation coefficients and p values in the correlation with AIM levels in the indicated tested item, in patients with or without hepatitis viral infection. n: sample number.
(DOCX)

Table S3 **Correlation coefficients between all variables that are candidates for determinant of AIM.** C: single regression coefficient, *p*: p-value. Cre: creatinine.
(DOCX)

Table S4 **AIM and various clinical markers in populations who exhibit very high or very low AIM levels.** Number of samples (n), and the correlation coefficients and p values in the correlation with AIM levels in identical parameter. Low: patients who exhibited less than 3.0 µg/ml of AIM, High: patients who exhibited more than 10.0 µg/ml of AIM.
(DOCX)
