# Supplementary figures and images for: Circulating AIM as an Indicator of Liver Damage and Hepatocellular Carcinoma in Humans
Source: PLoS One. 2014 Oct 10;9(10):e109123. doi: 10.1371/journal.pone.0109123 (PMC4193837; doi:10.1371/journal.pone.0109123)

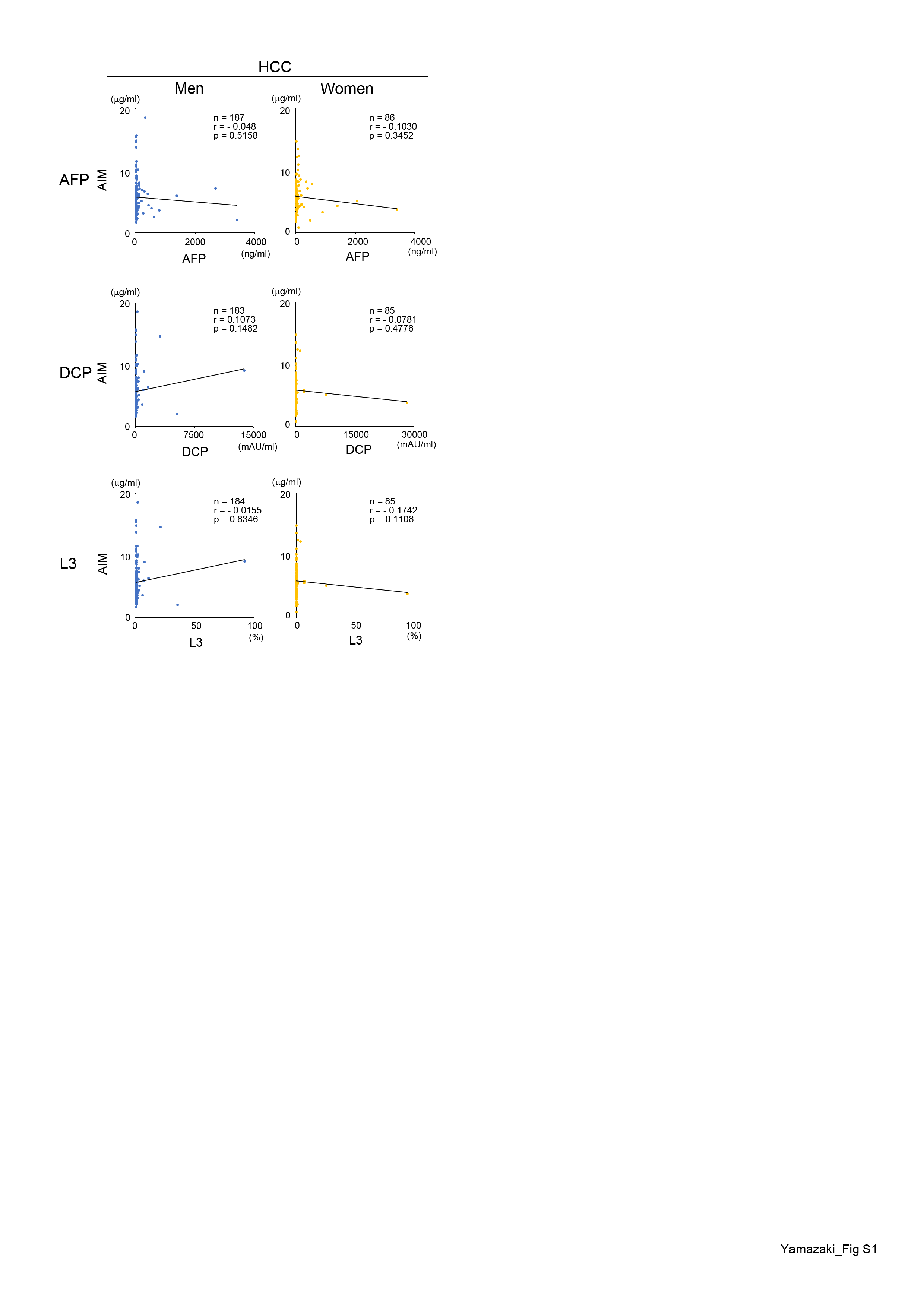

Supplement: Figure S1 — Correlation between AIM levels and various HCC markers. Men: blue dots, women: yellow dots. In HCC patients, no significant correlation was observed in levels of AIM and either HCC marker. (TIF) [file pone.0109123.s001.tif]

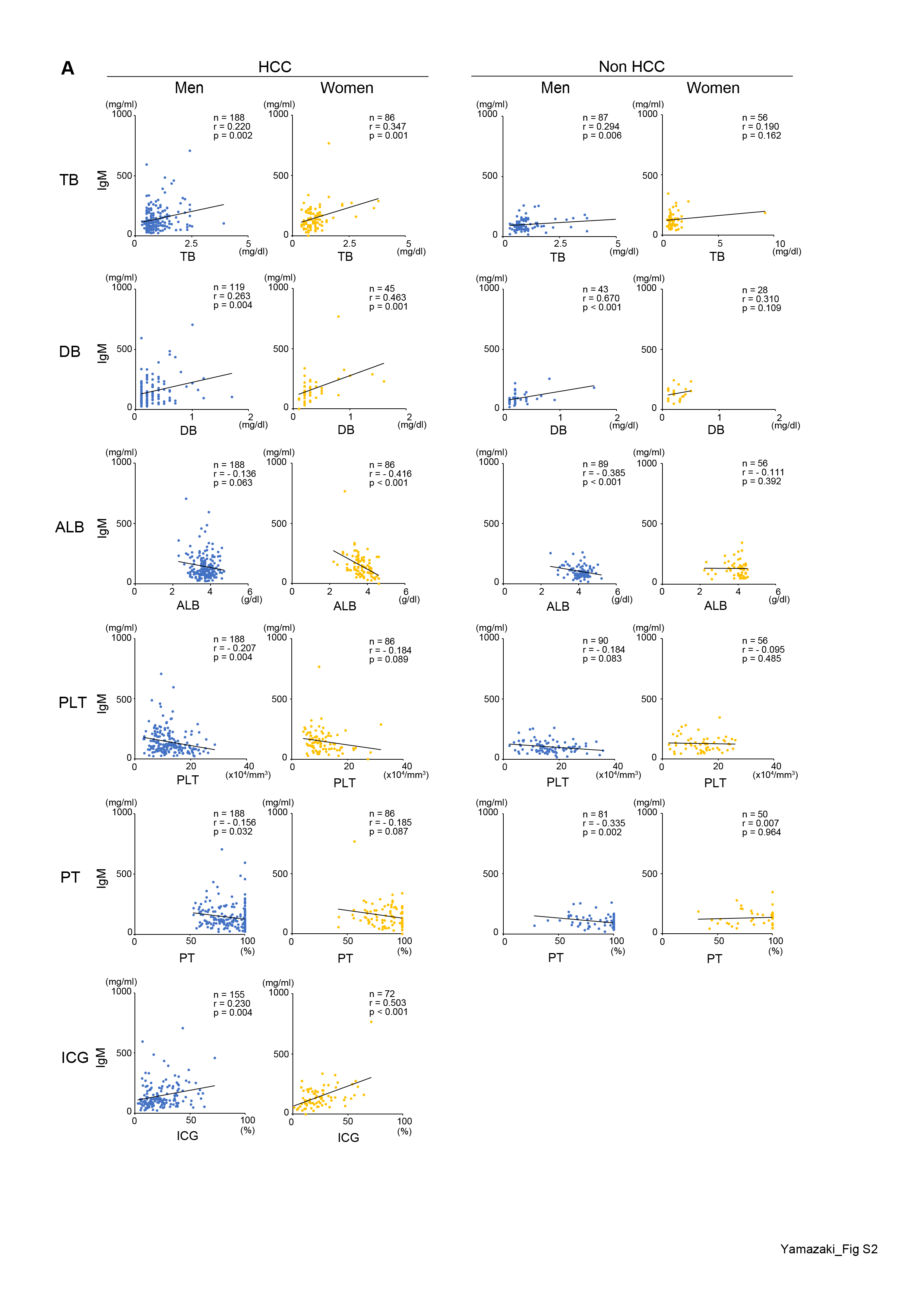

Supplement: Figure S2 — Correlation between IgM levels and various biomarkers representing liver function. Men: blue dots, women: yellow dots. ICG score was only available in HCC patients. (TIF) [file pone.0109123.s002.tif]

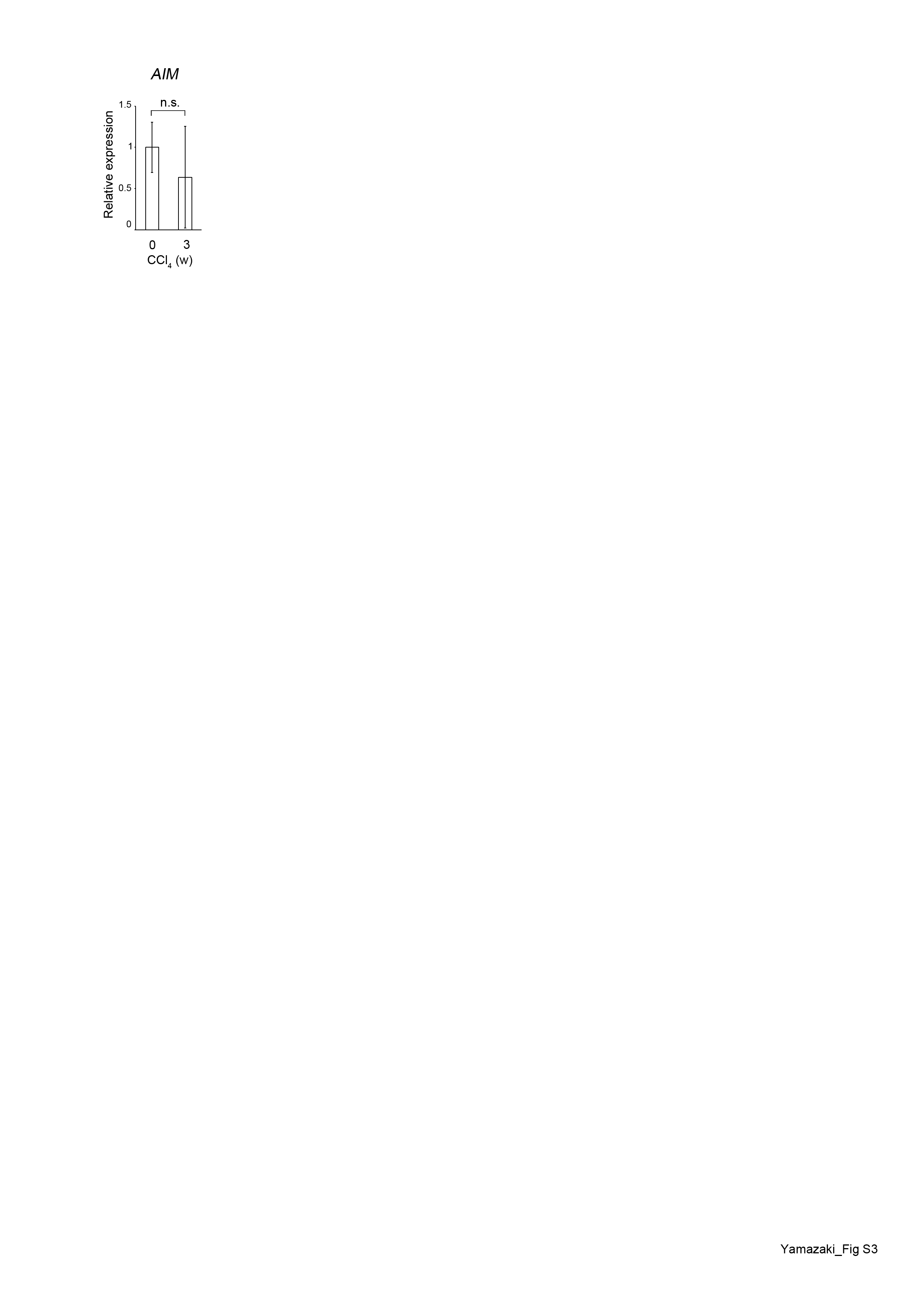

Supplement: Figure S3 — AIM expression did not increase in the liver in response to CCl4. mRNA levels of AIM in the liver from wild-type mice after administration of CCl4 for 3 wk. n = 3 for each. Error bar: SEM. (TIF) [file pone.0109123.s003.tif]
