# Supplementary material for: Circulating AIM as an Indicator of Liver Damage and Hepatocellular Carcinoma in Humans
Source: PLoS One. 2014 Oct 10;9(10):e109123. doi: 10.1371/journal.pone.0109123 (PMC4193837; doi:10.1371/journal.pone.0109123)
Supplement: Table S1 — Correlation in AIM level and different clinical parameters. Number of samples, and the correlation coefficients and p values in the correlation with AIM levels in separate tested item. n: sample numbers. (DOCX) [file pone.0109123.s004.docx]

|  | Men | | | | Women | | | | |
| --- | --- | --- | --- | --- | --- | --- | --- | --- | --- |
| Clinical parameters | n | correlation coefficient | | p value | n | correlation coefficient | | | p value |
| Age | 4055 | -0.050095 | 0.001419 | | 4225 | | -0.220057 | 1.69E-47 | |
| Height | 4051 | 0.0111226 | 0.479115 | | 4217 | | 0.0984 | 1.52E-10 | |
| Weight | 4051 | 0.0175232 | 0.26483 | | 4217 | | 0.0078769 | 0.609093 | |
| Body mass index (BMI) | 4051 | 0.0155529 | 0.322341 | | 4217 | | -0.040025 | 0.009338 | |
| Obesity index | 4051 | 0.0158366 | 0.313593 | | 4217 | | -0.039829 | 0.009691 | |
| % of fat mass | 2573 | 0.0069684 | 0.723862 | | 2936 | | -0.051849 | 0.004952 | |
| Waist circumference | 3954 | 0.0137148 | 0.388598 | | 4094 | | -0.066763 | 1.91E-05 | |
| 5m naked vision right | 3911 | 0.0115166 | 0.471512 | | 3773 | | -0.008344 | 0.608404 | |
| 5m naked vision left | 3911 | 0.005245 | 0.742983 | | 3772 | | -0.007093 | 0.663197 | |
| 5m corrected vision right | 2480 | 0.0171516 | 0.393231 | | 2307 | | 0.0481793 | 0.020657 | |
| 5m corrected vision left | 2480 | 0.0144285 | 0.472629 | | 2317 | | 0.0171946 | 0.408079 | |
| Pulmonary capacity | 801 | 0.0708896 | 0.044888 | | 771 | | 0.1016867 | 0.004709 | |
| Forced pulmonary capacity | 757 | 0.0932538 | 0.010255 | | 737 | | 0.1083964 | 0.003215 | |
| % pulmonary capacity | 757 | 0.0170801 | 0.638932 | | 1474 | | -0.04657 | 0.073871 | |
| Forced expiratory volume 1 sec (FEV1) | 801 | 0.0824845 | 0.019554 | | 771 | | 0.1519582 | 2.26E-05 | |
| Forced expiratory volume 1 sec % (FEV 1%) | 801 | -0.005916 | 0.867235 | | 771 | | 0.1024478 | 0.004406 | |
| Systolic blood pressure (1st time) | 4055 | -0.003319 | 0.832649 | | 4225 | | -0.091474 | 2.58E-09 | |
| Systolic blood pressure (2nd time) | 4055 | 0.002207 | 0.888267 | | 4225 | | -0.092735 | 1.55E-09 | |
| Diastolic blood pressure (1st time) | 4055 | -0.030721 | 0.050451 | | 4225 | | -0.090688 | 3.52E-09 | |
| Diastolic blood pressure (2nd time) | 4055 | -0.030502 | 0.052115 | | 4225 | | -0.081794 | 1.02E-07 | |
| Urinary specific gravity | 747 | 0.0642958 | 0.079061 | | 612 | | 0.0624547 | 0.122736 | |
| Urine pH | 684 | -0.034633 | 0.365786 | | 569 | | 0.0242064 | 0.564462 | |
| Cardio-thoracic ratio | 3858 | 0.0099599 | 0.536276 | | 3797 | | -0.115923 | 7.78E-13 | |
| Heart rate | 2970 | 0.0240514 | 0.190066 | | 3477 | | 0.0054472 | 0.748146 | |
| Red blood cell count | 4037 | 0.1050308 | 2.23E-11 | | 4019 | | 0.0853468 | 6E-08 | |
| Hemoglobin content | 4036 | -0.003709 | 0.813761 | | 4016 | | -0.054852 | 0.000506 | |
| White blood cell count | 3732 | 0.0120348 | 0.462349 | | 3567 | | 0.0042694 | 0.7988 | |
| Hematocrit | 3511 | -0.011223 | 0.506203 | | 3679 | | -0.044169 | 0.007374 | |
| Platelet count | 2369 | -0.029858 | 0.146273 | | 2573 | | 0.019201 | 0.330264 | |
| Mean corpuscular volume (MCV) | 667 | -0.059525 | 0.12459 | | 494 | | -0.142936 | 0.001446 | |
| Mean corpuscular hemoglobin (MCH) | 659 | -0.046293 | 0.235322 | | 488 | | -0.12847 | 0.004476 | |
| Mean corpuscular hemoglobin concentration (MCHC) | 657 | 0.0688813 | 0.077682 | | 484 | | -0.03428 | 0.451798 | |
| Neutrophil | 167 | 0.1603921 | 0.038398 | | 79 | | 0.1794403 | 0.113569 | |
| Lymphocyte | 168 | -0.175039 | 0.023246 | | 80 | | -0.241853 | 0.030668 | |
| Monocyte | 168 | -0.025392 | 0.743881 | | 80 | | 0.1022626 | 0.366718 | |
| Eosinophil | 168 | 0.0254994 | 0.742839 | | 80 | | 0.1316616 | 0.244359 | |
| Basophil | 168 | 0.0254989 | 0.742844 | | 80 | | 0.0630275 | 0.57861 | |
| Total serum protein | 993 | 0.2024664 | 1.2E-10 | | 852 | | 0.1372592 | 5.83E-05 | |
| Albumin (ALB) | 874 | 0.0098703 | 0.770753 | | 844 | | 0.0049866 | 0.884983 | |
| Albumin/globulin (A/G) | 331 | -0.178226 | 0.001128 | | 432 | | -0.153876 | 0.001336 | |
| Total bilirubin (TB) | 956 | -0.033528 | 0.300385 | | 608 | | 0.0033708 | 0.933895 | |
| Aspartate transaminase (AST) | 4054 | 0.0549249 | 0.000468 | | 4225 | | -0.047262 | 0.00212 | |
| Alanine transaminase (ALT) | 4055 | 0.0536685 | 0.000629 | | 4225 | | -0.030446 | 0.047831 | |
| Gamma-glutamyl transpeptidase (γ-GTP) | 4055 | 0.0189954 | 0.226531 | | 4225 | | -0.037634 | 0.01443 | |
| Alkaline phosphatase (ALP) | 2115 | 0.0946085 | 1.31E-05 | | 1415 | | -0.01512 | 0.569847 | |
| Lactate dehydrogenase (LDH) | 492 | 0.0095023 | 0.833481 | | 305 | | 0.0121631 | 0.832454 | |
| Leucyl aminopeptidase (LAP) | 39 | -0.136059 | 0.408864 | | 40 | | -0.04668 | 0.774858 | |
| Cholinesterase | 117 | 0.0261352 | 0.779702 | | 89 | | 0.0948383 | 0.376669 | |
| Creatine phosphokinase (CPK) | 35 | -0.112314 | 0.520638 | | 36 | | 0.0845583 | 0.623904 | |
| Serum amylase | 652 | 0.0333565 | 0.395138 | | 313 | | 0.0121195 | 0.830885 | |
| Total cholesterol | 3138 | -0.009465 | 0.596111 | | 2951 | | -0.09321 | 3.93E-07 | |
| HDL cholesterol | 4051 | -0.095976 | 9.31E-10 | | 4216 | | -0.058803 | 0.000133 | |
| LDL cholesterol | 4049 | 0.0221294 | 0.15917 | | 4214 | | -0.060345 | 8.87E-05 | |
| Triglyceride | 4048 | 0.049813 | 0.001523 | | 4216 | | -0.041874 | 0.006542 | |
| Creatinine | 2795 | 0.032652 | 0.084361 | | 2187 | | 0.0139582 | 0.51413 | |
| Urea nitrogen | 558 | 0.0571219 | 0.177848 | | 718 | | -0.033381 | 0.371774 | |
| Serum uric acid | 2998 | 0.0623188 | 0.00064 | | 2296 | | -0.049825 | 0.016956 | |
| Fasting blood sugar (FBS) | 3855 | 0.0293914 | 0.06805 | | 3899 | | -0.079064 | 7.69E-07 | |
| Blood sugar | 180 | 0.1406178 | 0.059726 | | 267 | | -0.081312 | 0.1853 | |
| HbA1c | 2406 | 0.0197599 | 0.332628 | | 3146 | | -0.125717 | 1.48E-12 | |
| Blood sugar 0 min | 107 | 0.149281 | 0.12487 | | 32 | | 0.0032415 | 0.985952 | |
| Blood sugar 60 min | 104 | 0.0075039 | 0.939736 | | 32 | | -0.056717 | 0.757836 | |
| Blood sugar 120 min | 104 | 0.1454336 | 0.140736 | | 32 | | 0.1049765 | 0.567461 | |
| Serum iron | 642 | 0.0007261 | 0.985351 | | 423 | | -0.012204 | 0.802389 | |
| C-reactive protein (CRP) | 773 | 0.0801841 | 0.025792 | | 521 | | 0.0255656 | 0.560406 | |
| Bone density % age matched | 218 | -0.054697 | 0.421653 | | 453 | | 0.1606006 | 0.000601 | |

**Table S1.** **Correlation in AIM level and different clinical parameters.** Number of samples, and the correlation coefficients and p values in the correlation with AIM levels in separate tested item. n: sample numbers.
