# Supplementary material for: Circulating AIM as an Indicator of Liver Damage and Hepatocellular Carcinoma in Humans
Source: PLoS One. 2014 Oct 10;9(10):e109123. doi: 10.1371/journal.pone.0109123 (PMC4193837; doi:10.1371/journal.pone.0109123)
Supplement: Table S2 — Correlation between AIM and liver function in patients with or without hepatitis viral infection. Number of samples, and the correlation coefficients and p values in the correlation with AIM levels in the indicated tested item, in patients with or without hepatitis viral infection. n: sample number. (DOCX) [file pone.0109123.s005.docx]

|  |  | Men | | | Women | | |
| --- | --- | --- | --- | --- | --- | --- | --- |
|  |  | correlation coefficient | n | p value | correlation coefficient | n | p value |
| TB | Virus (+) | 0.50448 | 209 | 6.81E-15 | 0.28399 | 108 | 0.002896 |
|  | Virus (-) | 0.57518 | 66 | 4.39E-07 | 0.50706 | 34 | 0.002209 |
| DB | Virus (+) | 0.46911 | 113 | 1.6E-07 | 0.39109 | 49 | 0.005459 |
|  | Virus (-) | 0.58770 | 49 | 9E-06 | 0.48669 | 19 | 0.034586 |
| ALB | Virus (+) | -0.45460 | 210 | 4.17E-12 | -0.59877 | 108 | 7.67E-12 |
|  | Virus (-) | -0.35020 | 67 | 0.00367 | -0.44958 | 34 | 0.00764 |
| PLT | Virus (+) | -0.39534 | 211 | 2.64E-09 | -0.43724 | 108 | 2.23E-06 |
|  | Virus (-) | -0.54318 | 67 | 2.04E-06 | -0.29812 | 34 | 0.0868 |
| PT | Virus (+) | 0.53862 | 203 | 1.14E-16 | -0.36301 | 97 | 0.000258 |
|  | Virus (-) | -0.52661 | 66 | 5.57E-06 | -0.42315 | 33 | 0.014144 |

**Table S2**. **Correlation between AIM and liver function in patients with or without hepatitis viral infection.** Number of samples, and the correlation coefficients and p values in the correlation with AIM levels in the indicated tested item, in patients with or without hepatitis viral infection. n: sample number.
