# Supplementary material for: Circulating AIM as an Indicator of Liver Damage and Hepatocellular Carcinoma in Humans
Source: PLoS One. 2014 Oct 10;9(10):e109123. doi: 10.1371/journal.pone.0109123 (PMC4193837; doi:10.1371/journal.pone.0109123)
Supplement: Table S3 — Correlation coefficients between all variables that are candidates for determinant of AIM. C: single regression coefficient, p: p-value. Cre: creatinine. (DOCX) [file pone.0109123.s006.docx]

| HCC Men |  | Age | TB | ALB | AST | ALT | PLT | PT | Cre |
| --- | --- | --- | --- | --- | --- | --- | --- | --- | --- |
| IgM | C | -0.0974 | 0.220 | -0.136 | -0.00377 | -0.0659 | -0.207 | -0.156 | 0.0115 |
|  | *p* | *0.184* | *0.002* | *0.063* | *0.959* | *0.369* | *0.004* | *0.032* | *0.880* |
| Age | C |  | -0.142 | -0.114 | -0.0305 | -0.0891 | 0.165 | 0.107 | 0.0776 |
|  | *p* |  | *0.052* | *0.118* | *0.677* | *0.224* | *0.024* | *0.145* | *0.310* |
| TB | C |  |  | -0.352 | 0.131 | -0.0716 | -0.426 | -0.270 | -0.149 |
|  | *p* |  |  | *<0.001* | *0.073* | *0.329* | *<0.001* | *<0.001* | *0.051* |
| ALB | C |  |  |  | -0.305 | -0.111 | 0.333 | 0.259 | -0.0811 |
|  | *p* |  |  |  | *<0.001* | *0.128* | *<0.001* | *<0.001* | *0.289* |
| AST | C |  |  |  |  | 0.853 | -0.276 | -0.143 | -0.0692 |
|  | *p* |  |  |  |  | *<0.001* | *<0.001* | *0.050* | *0.365* |
| ALT | C |  |  |  |  |  | -0.152 | -0.149 | -0.0518 |
|  | *p* |  |  |  |  |  | *0.037* | *0.041* | *0.499* |
| PLT | C |  |  |  |  |  |  | 0.176 | -0.0209 |
|  | *p* |  |  |  |  |  |  | *0.016* | *0.785* |
| PT | C |  |  |  |  |  |  |  | 0.00716 |
|  | *p* |  |  |  |  |  |  |  | *0.926* |
| HCC Women |  | Age | TB | ALB | AST | ALT | PLT | PT | Cre |
| IgM | C | -0.128 | 0.347 | -0.416 | 0.414 | 0.203 | -0.184 | -0.185 | 0.0131 |
|  | *p* | *0.240* | *0.001* | *<0.001* | *<0.001* | *0.061* | *0.089* | *0.087* | *0.911* |
| Age | C |  | -0.160 | -0.0571 | -0.166 | -0.148 | 0.193 | 0.223 | 0.231 |
|  | *p* |  | *0.142* | *0.601* | *0.126* | *0.175* | *0.075* | *0.039* | *0.046* |
| TB | C |  |  | -0.266 | 0.207 | 0.0236 | -0.118 | -0.295 | -0.126 |
|  | *p* |  |  | *0.013* | *0.0560* | *0.829* | *0.281* | *0.006* | *0.282* |
| ALB | C |  |  |  | -0.177 | -0.0106 | 0.317 | 0.302 | 0.098 |
|  | *p* |  |  |  | *0.103* | *0.922* | *0.003* | *0.004* | *0.403* |
| AST | C |  |  |  |  | 0.898 | -0.199 | -0.108 | -0.196 |
|  | *p* |  |  |  |  | *<0.001* | *0.066* | *0.322* | *0.091* |
| ALT | C |  |  |  |  |  | -0.208 | -0.0170 | -0.251 |
|  | *p* |  |  |  |  |  | *0.054* | *0.876* | *0.030* |
| PLT | C |  |  |  |  |  |  | 0.0594 | 0.122 |
|  | *p* |  |  |  |  |  |  | *0.587* | *0.299* |
| PT | C |  |  |  |  |  |  |  | 0.120 |
|  | *p* |  |  |  |  |  |  |  | *0.306* |
| Non-HCC Men |  | Age | TB | ALB | AST | ALT | PLT | PT | Cre |
| IgM | C | -0.0981 | 0.188 | -0.256 | 0.0957 | 0.0527 | -0.224 | -0.284 | -0.0670 |
|  | *p* | *0.361* | *0.077* | *0.015* | *0.372* | *0.624* | *0.034* | *0.011* | *0.596* |
| Age | C |  | 0.0908 | -0.330 | -0.174 | -0.309 | -0.258 | -0.0493 | -0.0903 |
|  | *p* |  | *0.398* | *0.002* | *0.103* | *0.003* | *0.015* | *0.664* | *0.474* |
| TB | C |  |  | -0.409 | -0.0101 | -0.159 | -0.535 | -0.569 | -0.175 |
|  | *p* |  |  | *<0.001* | *0.926* | *0.137* | *<0.001* | *<0.001* | *0.164* |
| ALB | C |  |  |  | -0.1203 | 0.0447 | 0.409 | 0.502 | 0.0247 |
|  | *p* |  |  |  | *0.261* | *0.678* | *<0.001* | *<0.001* | *0.845* |
| AST | C |  |  |  |  | 0.851 | -0.0533 | -0.0909 | -0.172 |
|  | *p* |  |  |  |  | *<0.001* | *0.620* | *0.423* | *0.171* |
| ALT | C |  |  |  |  |  | 0.175 | 0.0774 | -0.118 |
|  | *p* |  |  |  |  |  | *0.101* | *0.495* | *0.349* |
| PLT | C |  |  |  |  |  |  | 0.520 | -0.0908 |
|  | *p* |  |  |  |  |  |  | *<0.001* | *0.472* |
| PT | C |  |  |  |  |  |  |  | 0.0836 |
|  | *p* |  |  |  |  |  |  |  | *0.540* |
| Non-HCC Women |  | Age | TB | ALB | AST | ALT | PLT | PT | Cre |
| IgM | C | -0.161 | 0.152 | -0.0122 | 0.187 | 0.0671 | -0.0491 | 0.0564 | -0.0946 |
|  | *p* | *0.235* | *0.263* | *0.929* | *0.168* | *0.623* | *0.719* | *0.716* | *0.546* |
| Age | C |  | 0.0641 | -0.156 | 0.219 | 0.0659 | -0.136 | -0.0805 | 0.0943 |
|  | *p* |  | *0.639* | *0.252* | *0.105* | *0.629* | *0.317* | *0.604* | *0.547* |
| TB | C |  |  | -0.423 | 0.167 | -0.0847 | 0.0192 | -0.448 | 0.0542 |
|  | *p* |  |  | *0.001* | *0.218* | *0.535* | *0.888* | *0.002* | *0.730* |
| ALB | C |  |  |  | -0.0334 | 0.257 | 0.381 | 0.667 | -0.013 |
|  | *p* |  |  |  | *0.807* | *0.056* | *0.004* | *<0.001* | *0.936* |
| AST | C |  |  |  |  | 0.770 | -0.198 | -0.0745 | -0.130 |
|  | *p* |  |  |  |  | *<0.001* | *0.144* | *0.631* | *0.406* |
| ALT | C |  |  |  |  |  | 0.0399 | 0.160 | -0.127 |
|  | *p* |  |  |  |  |  | *0.771* | *0.299* | *0.418* |
| PLT | C |  |  |  |  |  |  | 0.461 | -0.120 |
|  | *p* |  |  |  |  |  |  | *0.002* | *0.443* |
| PT | C |  |  |  |  |  |  |  | -0.494 |
|  | *p* |  |  |  |  |  |  |  | *0.004* |

**Table S3.** **Correlation coefficients between all variables that are candidates for determinant of AIM.** C: single regression coefficient, *p*: p-value. Cre: creatinine.
