# Supplementary material for: Circulating AIM as an Indicator of Liver Damage and Hepatocellular Carcinoma in Humans
Source: PLoS One. 2014 Oct 10;9(10):e109123. doi: 10.1371/journal.pone.0109123 (PMC4193837; doi:10.1371/journal.pone.0109123)
Supplement: Table S4 — AIM and various clinical markers in populations who exhibit very high or very low AIM levels. Number of samples (n), and the correlation coefficients and p values in the correlation with AIM levels in identical parameter. Low: patients who exhibited less than 3.0 µg/ml of AIM, High: patients who exhibited more than 10.0 µg/ml of AIM. (DOCX) [file pone.0109123.s007.docx]

**Men**

| AIM | Low | | | | | High | | | |
| --- | --- | --- | --- | --- | --- | --- | --- | --- | --- |
| Clinical parameters | correlation coefficient | | n | p value | | correlation coefficient | | n | p value |
| Age (year) | 0.181833 | 18 | | | 0.470219 | 0.153916 | 13 | | 0.615636 |
| BMI (μg/ml) | -0.029217 | 18 | | | 0.908380 | -0.029005 | 13 | | 0.925062 |
| IgM (mg/dl) | -0.136546 | 18 | | | 0.589013 | 0.333393 | 13 | | 0.265632 |
| AST (U/L) | -0.149472 | 18 | | | 0.553870 | 0.370970 | 13 | | 0.212065 |
| ALT (U/L) | 0.025571 | 18 | | | 0.919777 | 0.101878 | 13 | | 0.740510 |
| TB (mg/dl) | 0.021083 | 12 | | | 0.948146 | 0.313632 | 11 | | 0.347630 |
| DB (mg/dl) | 0.024670 | 18 | | | 0.922595 | -0.691102 | 13 | | 0.008897 |
| ALB (g/dl) | -0.334580 | 18 | | | 0.174751 | -0.550700 | 13 | | 0.051132 |
| PLT (x10^4^/mm^3^) | -0.111299 | 18 | | | 0.660174 | 0.169686 | 13 | | 0.579434 |
| PT (%) | 0.062361 | 15 | | | 0.825260 | 0.679786 | 13 | | 0.010582 |
| Cre (mg/dl) | -0.070269 | 12 | | | 0.828206 | 0.099497 | 13 | | 0.746393 |
| ICG (%) | 0.181833 | 18 | | | 0.470219 | 0.153916 | 13 | | 0.615636 |

**Women**

| AIM | Low | | | | | High | | | | |
| --- | --- | --- | --- | --- | --- | --- | --- | --- | --- | --- |
| Clinical parameters | correlation coefficient | | n | | p value | correlation coefficient | | n | p value | |
| Age (year) | 0.001576 | 8 | | 0.997045 | | -0.261267 | 6 | | | 0.617016 |
| BMI (μg/ml) | 0.190895 | 8 | | 0.650673 | | 0.140346 | 6 | | | 0.790863 |
| IgM (mg/dl) | -0.390196 | 8 | | 0.339251 | | -0.026476 | 6 | | | 0.960295 |
| AST (U/L) | -0.563536 | 8 | | 0.145762 | | -0.082256 | 6 | | | 0.876895 |
| ALT (U/L) | -0.002266 | 8 | | 0.995751 | | 0.848577 | 6 | | | 0.032658 |
| TB (mg/dl) | -0.262707 | 3 | | 0.830770 | | 0.788493 | 5 | | | 0.112990 |
| DB (mg/dl) | -0.067884 | 8 | | 0.873108 | | -0.056880 | 6 | | | 0.914772 |
| ALB (g/dl) | 0.173042 | 8 | | 0.681965 | | 0.002223 | 6 | | | 0.996666 |
| PLT (x10^4^/mm^3^) | -0.316153 | 8 | | 0.445529 | | -0.834015 | 6 | | | 0.039040 |
| PT (%) | 0.515298 | 6 | | 0.295467 | | -0.300988 | 6 | | | 0.562151 |
| Cre (mg/dl) | 0.067556 | 7 | | 0.885575 | | 0.633570 | 6 | | | 0.176806 |
| ICG (%) | -0.189744 | 8 | | 0.652676 | | -0.261267 | 6 | | | 0.617016 |

**Table S4. AIM and various clinical markers in populations who exhibit very high or very low AIM levels.** Number of samples (n), and the correlation coefficients and p values in the correlation with AIM levels in identical parameter. Low: patients who exhibited less than 3.0 μg/ml of AIM, High: patients who exhibited more than 10.0 μg/ml of AIM.
